# Intestinal microflora promotes Th2-mediated immunity through NLRP3 in damp and heat environments

**DOI:** 10.3389/fimmu.2024.1367053

**Published:** 2024-05-02

**Authors:** Yi Luo, Xinhua Huang, Haiying Hu, Yao Wang, Xiangrong Feng, Song Chen, Huanhuan Luo

**Affiliations:** ^1^ State Key Laboratory of Traditional Chinese Medicine Syndrome, Guangzhou University of Chinese Medicine, Guangzhou, China; ^2^ School of Basic Medical Sciences, Guangzhou University of Chinese Medicine, Guangzhou, China; ^3^ West China Hospital, Sichuan University, Chengdu, China; ^4^ Science and Technology Innovation Center, Guangzhou University of Chinese Medicine, Guangzhou, China

**Keywords:** damp-heat environment, NLRP3, Th2-mediated immunity, intestinal microflora, transcriptome

## Abstract

**Background:**

With the worsening of the greenhouse effect, the correlation between the damp-heat environment (DH) and the incidence of various diseases has gained increasing attention. Previous studies have demonstrated that DH can lead to intestinal disorders, enteritis, and an up-regulation of NOD-like receptor protein 3 (NLRP3). However, the mechanism of NLRP3 in this process remains unclear.

**Methods:**

We established a DH animal model to observe the impact of a high temperature and humidity environment on the mice. We sequenced the 16S rRNA of mouse feces, and the RNA transcriptome of intestinal tissue, as well as the levels of cytokines including interferon (IFN)-γ and interleukin (IL)-4 in serum.

**Results:**

Our results indicate that the intestinal macrophage infiltration and the expression of inflammatory genes were increased in mice challenged with DH for 14 days, while the M2 macrophages were decreased in *Nlrp3* ^-/-^ mice. The alpha diversity of intestinal bacteria in *Nlrp3* ^-/-^ mice was significantly higher than that in control mice, including an up-regulation of the *Firmicutes/Bacteroidetes* ratio. Transcriptomic analysis revealed 307 differentially expressed genes were decreased in *Nlrp3* ^-/-^ mice compared with control mice, which was related to humoral immune response, complement activation, phagocytic recognition, malaria and inflammatory bowel disease. The ratio of IFN-γ/IL-4 was decreased in control mice but increased in *Nlrp3* ^-/-^ mice.

**Conclusions:**

Our study found that the inflammation induced by DH promotes Th2-mediated immunity via NLRP3, which is closely related to the disruption of intestinal flora.

## 1 Introduction

In 2019, The World Meteorological Organization (WMO) proposed that the temperature could rise by 3-5°C in this century (1). Most tropical and subtropical areas experience long summers and short winters with extended periods of sunshine. Studies have found that the incidence of respiratory, cardiovascular and renal diseases, as well as mortality in patients with cardiovascular disease (2), were closely related to low-intensity heatwaves in tropical and subtropical humid areas (3). The Lingnan region of China, located in the east Asian monsoon region, is characterized by a subtropical monsoon oceanic climate. Most areas in this region have long summers and short winters with prolonged periods of sunshine. In tropical or subtropical humid regions, there is a small temperature difference between day and night (4, 5). Even after escaping from the hot environment, a sustained hot climate can still cause prolonged effects on the body for 21 to 28 days (6, 7).

The damp-heat environment (DH) can significantly impact animals (8), leading to immunosuppression, weakened disease resistance, and stunted growth (9, 10). It also increases heat stress, morbidity and mortality (11). The body’s heat dissipation is closely linked to environmental temperature and humidity (12). High humidity can further intensify the effects of high temperature by inhibiting the body’s heat dissipation through evaporative cooling, which makes it difficult to regulate core body temperature (13). Consequently, the body compensates by undergoing metabolic reprogramming (14), which can affect hormone metabolism, heart rate, respiratory rate, skin and intestinal temperature, and gene expression patterns (15, 16). These changes can result in electrolyte imbalance, endocrine disorders, and immune suppression (17, 18).

Environmental changes can also impact the ecology and function of the intestines. Heat stress can cause injury to the intestinal barrier, leading to systemic inflammation (19). The composition of the gut microbiota is closely related to the metabolic and immune profiles of the host organisms (20, 21). Temperature changes disrupt the diversity of enterobacteria, making the host more susceptible to intestinal diseases (19–21).In a previous study, we found that mice exposed to DH showed mild enteritis (22). Furthermore, there have some studies showed the inflammatory response of certain chronic diseases was closely associated with the NOD-like receptor protein 3 (NLRP3) pathway (23), which regulates the immune inflammatory response through downstream cytokines (24). For instance, NLRP3 inflammasome activates inflammatory cytokines such as IL-1β and IL-18 (25). Macrophages can be phenotypically polarized by surrounding microenvironmental stimuli and signals to mount specific functional programs. NLRP3 inflammasome-driven inflammation recruits inflammatory cells including neutrophils and macrophages (26), which release cytokines that causes macrophage polarization (27), affecting the immune inflammatory response process.

The balanced ratio of immune cell subgroups, such as Th1/Th2 cell subgroups plays a key regulatory role and is also particularly relevant to the cellular immune and humoral immune systems of the body. Therefore, it is proposed that the activation of the NLRP3 pathway caused by intestinal dysbiosis may mediate the imbalance of the Th1/Th2 immune response, which may be closely associated with chronic diseases caused by DH. This study conducted tests on *Nlrp3* ^-/-^ mice subjected to DH intervention for 14 days, using the 16S rRNA, the transcriptome, and the protein microarray to explore the key roles of gut microbiota, NLRP3 and immune inflammation in DH stimulation.

## 2 Materials and methods

### 2.1 Animal and ethical approval

Male-specific pathogen-free (SPF) C57BL/6J mice and *Nlrp3* ^-/-^ mice (7 weeks of age) weighing 20 ± 2 g were purchased from Guangdong Medical Experimental Animal Center (China). This study was performed under the supervision and assessment of the Laboratory Animal Ethics and Welfare Committee (AEWC) of Zhongshan Hospital of Traditional Chinese Medicine (no. AEWC-2022062). All experimental procedures were performed by the recommendations of the National Institutes of Health Guide for the Care and Use of Laboratory Animals [National Research Council, Guide for the Care and Use of Laboratory Animals. (2011)].

### 2.2 Grouping and treatments

The mice were housed in the environment of SPF constant temperature (23 ± 2°C) and average humidity (55 ± 5%), with a 12-12 hour light/dark cycle. After sample size calculations, 20 SPF C57BL/6J mice were randomly assigned to two groups according to the random number table method: a normal environment group (C-NC), a high temperature and high humidity environment group (C-DH). 20 *Nlrp3* ^-/-^ mice were randomly divided into two groups as the same: a normal environment group (K-NC), a high temperature and high humidity environment group (K-DH).

Using an artificial climate box (model: RXZ-158A-LED, Ningbo Jiangnan Instrument Factory, China) to simulate high humidity and high-temperature environment. The DH group was exposed to 33 ± 2°C and 85 ± 5% relative humidity for two weeks. Take materials for evaluation on the day 14. All of the mice had free access to food and water and renewed daily.

### 2.3 Detection of intestinal macrophages in mice

Intestine tissue paraffin sections of mice from each group on the 14th day were taken and baked in a 65°C oven for 2 hours. They were then deparaffinized in xylene I and II for 10 minutes each, followed by immersion in 100%, 95%, 80%, and 75% alcohol for 5 minutes each. The sections were washed three times with PBS buffer, 5 minutes each time. The tissue sections were immersed in sodium citrate buffer and heated to boiling, then kept on medium heat for 10 min. After cooling, rinse three times with PBS as before, wipe the moisture around the tissue on the slice, and incubate in a humid box with 3% hydrogen peroxide solution for 15 min. Rinse three times with PBS as before, wipe the moisture around the tissue on the slice, and incubate in a humid box with 10% BSA solution for 20 min. After discarding the blocking solution, slightly air dry and add an appropriate amount of primary antibody working solution, and incubate overnight at 4°C in a refrigerator. Then the tissue slides were incubated consecutively with primary antibodies F4/80 (1:200, #70076, Cell Signaling, USA), CD11c (1:200, #97585, Cell Signaling, USA), CD206 (1:200, #24595, Cell Signaling, USA). Incubate with horseradish peroxidase-conjugated secondary antibody, rinse three times with PBS as before. Apply DAB to each section for 10 min, counterstain with hematoxylin, wash, and mount the slides. Observe the positive expression in each group of intestinal tissue under a microscope. The positive expression appears as brown-yellow particles. Each slice randomly selects 10 unified and fixed magnification fields for counting. Record the average as the positive data for macrophage immunohistochemistry in the mice’s intestinal tissue.

### 2.4 Mice intestinal microbiota detection

Using the CTAB/SDS method, extract total genomic DNA from fecal samples. Use 1% agarose gel to determine the concentration and purity of DNA samples. Based on the concentration results, dilute the DNA concentration uniformly to 1 ng/μl using DEPC water. Amplify the 16S rRNA gene using barcode-specific primers. All PCR reactions are carried out in a 30 μL reaction, with 15 μL of Phusion High-Fidelity PCR Master Mix (New England Biolabs), 0.2 μM forward and reverse primers, and approximately 10 ng of template DNA. The program is as follows: 98°C/1 min, 98°C/10 sec for 30 cycles, then 50°C/30 sec, 72°C/60 sec, 72°C/5 min. Mix the carrier buffer containing SYB Green with the PCR product in equal volumes and perform agarose gel electrophoresis on a 2% agarose gel. Select samples with suitable brightness for the main band (400-450 bp) for the next experiment. Mix the samples of equal density and purify the mixed PCR products using a AxyPrepDNA Gel Extraction Kit (AXYGEN). Quantify the PCR recovery products using a fluorescent quantitation system according to the preliminary quantification results shown by electrophoresis, and mix them according to the sequencing requirements of different samples (NEB Next^®^Ultra™DNA Library Prep Kit for Illumina, NEB, USA). Follow the manufacturer’s instructions and add the index codes according to the standard procedure. The library quality is evaluated using the Qubit@ 2.0 Fluorometer (Thermo Scientific) and Agilent Bioanalyzer system. Finally, the sample library is sequenced using the Illumina Miseq 600 platform to generate corresponding paired reads (250 bp).

Each segment of the original DNA fragment is paired-end read, and each sample is assigned paired-end reads based on the unique barcode of each fragment. OTU clustering and species annotation sequence analysis are performed using the UPARSE software package. The α diversity of the samples is analyzed using an internal Perl script. The same OTUs are assigned to a collection of sequences with a similarity of 97% or higher. The OTU table is rarefied, and two indices, including Shannon and Simpson indices, are calculated to measure α diversity. The relative abundance of bacterial diversity from phylum to species level is represented by a percentage bar chart.

### 2.5 Intestinal gene transcriptome

Total RNA extraction from the organization was performed. The A260/A280 absorbance ratio of the RNA samples was measured (Nanodrop ND-2000, Thermo Scientific, USA), and the RIN value of the RNA was determined to ensure the quality of the RNA samples included in the experiment (Agilent Bioanalyzer 4150, AgilentTechnologies, CA, USA). The PE library was prepared according to the instructions of the mRNA-seq Lib Prep Kit (ABclonal, China). Oligo (dT) magnetic beads were used to purify mRNA from 1μg of total RNA, followed by fragmentation of the mRNA using first strand synthesis buffer. Subsequently, using mRNA fragments as templates, the first strand of cDNA was synthesized using random primers and reverse transcriptase RNase H. Then, the second strand of cDNA was synthesized using DNA polymerase I, ribonuclease H, buffer, and dNTPs. The synthesized double-stranded cDNA fragments were ligated with adapter sequences and amplified using PCR. After quality inspection and evaluation of the purified PCR products, sequencing was performed using the PE150 read length sequencing platform.

Using the sequencing results generated data for bioinformatics analysis (Illumina). The raw data in Fastq format were processed using Perl scripts to remove adapter sequences, filter out low-quality data where the number of bases with a quality score of less than or equal to 25 accounted for 60% or more, and data with a proportion of undetermined bases (N) greater than 5%, to obtain qualified data for subsequent analysis. The qualified data were aligned to the reference genome using HISAT2 software (http://daehwankimlab.github.io/hisat2/) to obtain mapped reads for further analysis. Feature Counts (http://subread.sourceforge.net/) were used to calculate the mapping of reads to each gene and the FPKM value of each gene based on the gene length. DESeq2 (http://bioconductor.org/packages/release/bioc/html/DESeq2.html) was used to analyze the differential expression of genes between groups.

GO and KEGG enrichment analysis were performed on the differentially expressed genes to assess their functional enrichment and to explain the differences between samples at the gene functional level. The R package clusterProfiler was used to analyze and plot the results of GO functional enrichment and KEGG pathway enrichment.

### 2.6 Cytokines detected by protein microarray chip

The slide chip was taken out from the kit (QAM-INF-1-2, RayBiotech, Inc., USA) and allowed to equilibrate at room temperature for 20 to 30 minutes. The sealing package was torn open and the sealing strip was peeled off, and the chip was placed at room temperature to dry for 1 to 2 hours. 100 µL of sample dilution solution was added to each well and incubated at room temperature on a shaker for 1 hour to block the quantitative antibody chip. Cytokine standards were diluted in gradients according to the instructions, with 6 gradients and one negative control, and prepared for use. After removing the buffer from each well and air drying slightly, 100 µL of standard solution and sample dilution solution were added to the marked wells (100 µL of 2-fold diluted serum), and incubated overnight on a shaker at 4°C. First, dilute the 20× washing solution 1:20 with deionized water. Clean with the diluted 1× washing solution, adding 250µL to each well and shaking at high intensity for 10 seconds, repeating this process 10 times. During the waiting period, dilute the 20× washing solution for the second washing solution with deionized water. Then switch to the 1× washing solution for the second washing, adding 250µL to each well and shaking at high intensity for 10 seconds, repeating this process 6 times. After centrifuging the vial containing the antibody mixture for detection, add 1.4ml of sample diluent, mix well and centrifuge again. Add 80µL of detection antibody to each well and incubate at 37°C on a shaker for 2 hours. Follow the same washing steps as before. After centrifuging the vial containing Cy3-streptavidin, add 1.4ml of sample diluent, mix well and centrifuge again. Add 80µL of Cy3-streptavidin to each well, cover the slide with a light-shielding paper and incubate at 37°C on a shaker for 1 hour. Follow the same washing steps as before.

Remove the slide frame and use a laser scanner Cy3 or the green channel (excitation frequency=532nm) to collect data signals (InnoScan 300 Microarray Scanner, Innopsys, France). The raw data obtained from chip scanning is processed for background subtraction and inter-chip normalization using Raybiotech software. Take the average of the standard data and normalize it. Take the double logarithm and perform linear regression with R2>0.9, and list the data and graphs of the standard curve. Take the average of the data from all samples, normalize it, and calculate the concentration of each cytokine in each sample group based on the standard curves of each factor.

### 2.7 Statistical analysis

*One-way ANOVA* or *Kruskal-Wallis* multi-comparisons test was used to compare differences across the four experimental groups, and continued to use *Tukey’s method* or *Bonferroni correction* for further analysis (SPSS Statistics 26 software, IBM Software, USA). Data of 16S rRNA amplicon sequencing, transcriptomics, and protein microarray data were analyzed using *moderated t-statistics* and the *Benjamini Hochberg* method to control the false discovery rate. The STAMP software (http://kiwi.cs./Software/STAMP) and LEfSe (http://galaxy.biobakery.org/) were used to confirm differences in the abundances of individual taxonomy. DESeq2 (http://bioconductor.org/packages/release/bioc/html/DESeq2.html) was used for the statistical analysis of differential gene data. Statistical significance for all data was set at *P*<0.05. Data are presented as mean ± standard error of the mean (SEM).

## 3 Results

### 3.1 *Nlrp3* deficiency alleviates the characteristic changes of animals and reduces ileal M2 macrophages in DH

**Figure 1:**
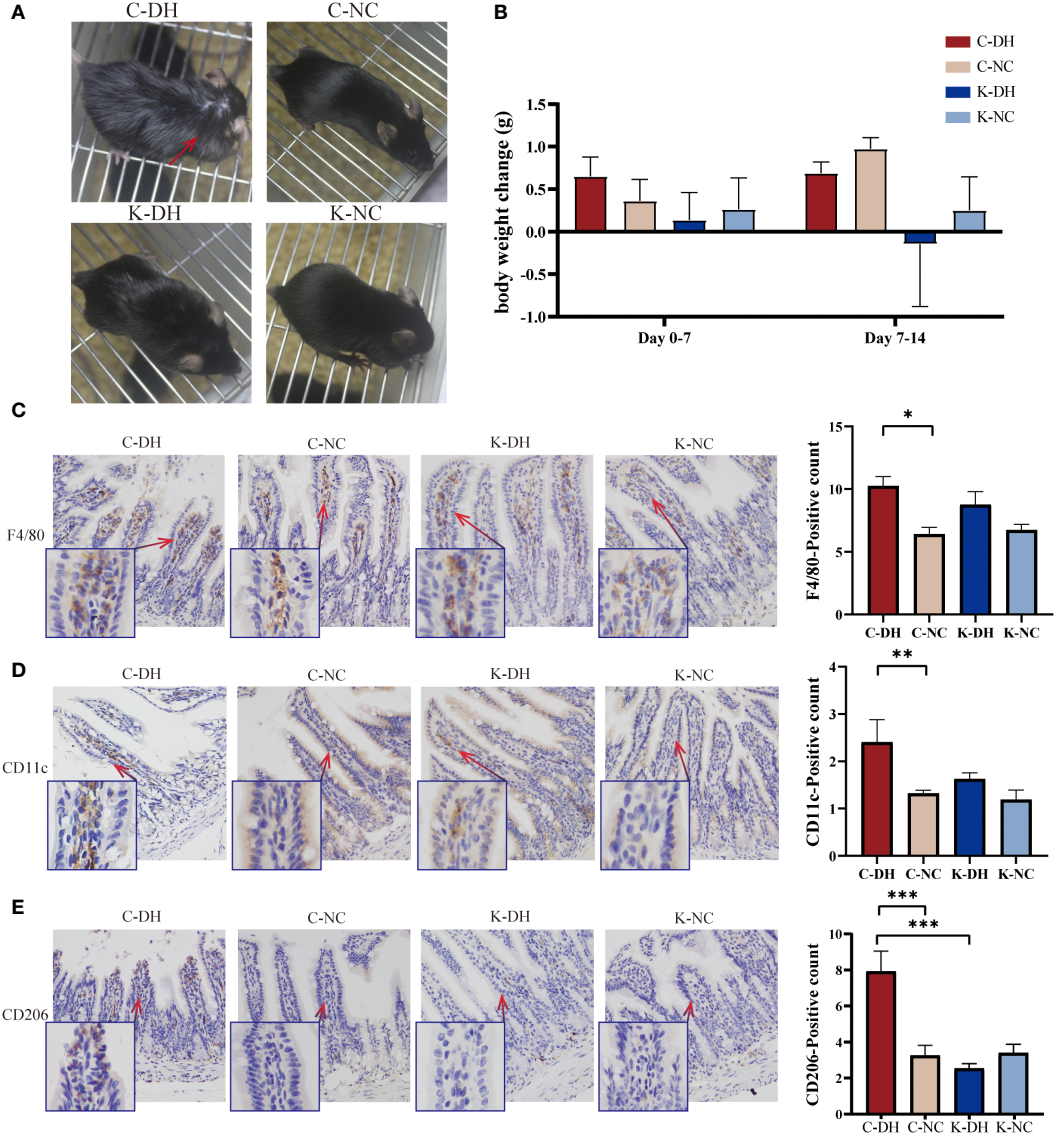
The characterization of mice and ileum macrophages in damp and heat environments (DH). **(A)** Fur changes in mice on day 14. **(B)** Weight changes in mice from each group. **(C-E)** Immunohistochemical staining of macrophages in intestinal tissues. C57 mice model of humid and hot environment (C-DH). C57 mice in the normal control environment (C-NC). *Nlrp3*-/- mice model of humid and hot environment (K-DH). *Nlrp3*-/- mice in the normal control environment (K-NC). F4/80 marking total macrophage count. CD11c marking M1 macrophages. CD206 marking M2 macrophages. *P<0.05. **P<0.01. ***P<0.001.

The DH environment modeling caused characteristic changes in control mice with the DH model (C-DH) group from the 14th day, such as increased tendency to be inactive, greasy and dirty fur (**Supplementary Figure S1A**), and decreased appetite for oily food (**Supplementary Figure S1B**). Compared to the characteristic changes observed in C-DH mice, *Nlrp3* ^-/-^ mice with the DH model (K-DH) group showed improved mental state and almost normal fur (**Figure 1A**). The body weight of the K-DH group was lower than that of the C-DH group (**Figure 1B**).

Using the immunohistochemistry method recognizes endogenous levels of the marker protein to detect intestinal macrophages. F4/80 marking total macrophage count. CD11c marking M1 macrophages. CD206 marking M2 macrophages. On the 14th day of modeling, the total number of macrophages in the ileum tissues of the C-DH group significantly increased (*P*<0.05), while the K-DH group had slightly fewer macrophages than the C-DH group (*P*>0.05) (**Figure 1C**). Both M1 (*P*<0.01) and M2 (*P*<0.001) macrophages in the C-DH group showed a significant increase (**Figures 1D, E**), while the positive expression of M2 macrophages in the ileum tissues of the K-DH group was significantly reduced compared to the C-DH group (*P*<0.001) (**Figure 1E**).

### 3.2 DH reduces gut microbiota structure and species diversity through *Nlrp3*

**Figure 2:**
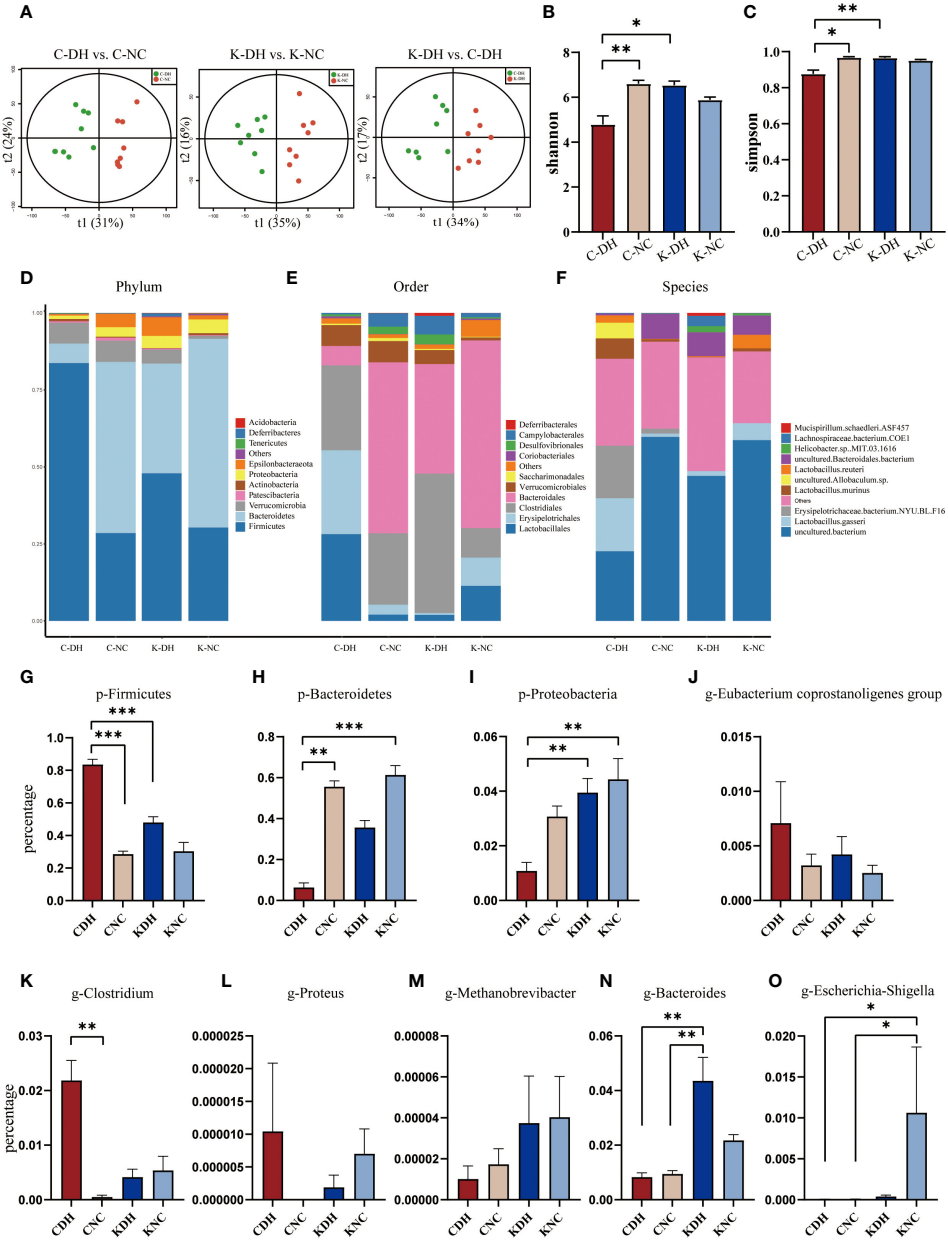
Differential gut microbiota induced by DH. **(A)** Principal Component Analysis of mice. **(B, C)** Shannon and Simpson index of intestinal microbiota in mice. **(D-F)** Comparison of phylum, class, and genus levels of intestinal microbiota in mice. **(G-O)** Comparison of species abundance of Firmicutes and other bacteria in mouse gut. C57 mice model of humid and hot environment (C-DH). C57 mice in the normal control environment (C-NC). *Nlrp3*-/- mice model of humid and hot environment (K-DH). *Nlrp3*-/- mice in the normal control environment (K-NC). *P<0.05. **P<0.01. ***P<0.001.

On the 14th day of modeling, Principal Component Analysis (PCA) shows that the inter-group clustering is well-distinguished (**Figure 2A**). In the C-DH group, the Shannon and Simpson indexes of gut microbiota significantly decreased (*P*<0.05), while the Shannon and Simpson indexes in the K-DH group remained at a higher level (**Figures 2B, C**).

The gut microbiota species structure can be affected by a DH, and *Nlrp3* plays an important role in this process. The ratio of *Firmicutes*/*Bacteroidetes* was increased in the C-DH group (**Figures 2D, G, H**, **Supplementary Table S1**), while *Proteobacteria* was slightly increased in *Nlrp3* ^-/-^ mice (**Figures 2D, I**, **Supplementary Table S1**). The C-DH group of mice had fewer *Bacteroidales*, while *Nlrp3* ^-/-^ mice had more *Bacteroidales* (**Figure 2E**). The K-DH group had more *Clostridiales* in the gut compared to the other three groups (**Figure 2E**). The genus *Lactobacillus* was significantly reduced in the K-DH group (**Figure 2F**). The genera *Clostridium*, *Proteus*, and *Eubacterium* showed an increasing trend in the C-DH group, but there was no significant decrease in the K-DH group (**Figures 2J-L**, **Supplementary Table S2**). *Methanobrevibacter*, *Bacteroides*, and *Escherichia* increased in the K-DH group compared to the C-DH group (**Figures 2M-O**, **Supplementary Table S2**).

The Cluster of Orthologous Groups (COG) of gut microbiota are mainly enriched in four primary functions: cellular processes and signaling, information storage and processing, metabolism, and unidentified functions. These four primary functions encompassed 25 secondary functions. The gut microbiota in the K-DH group was associated with carbohydrate transport, and metabolism, energy production and conversion and metabolism. The gut microbiota in the C-DH group was associated with nucleotide transport and metabolism (**Supplementary Figure S2**).

**Figure 3:**
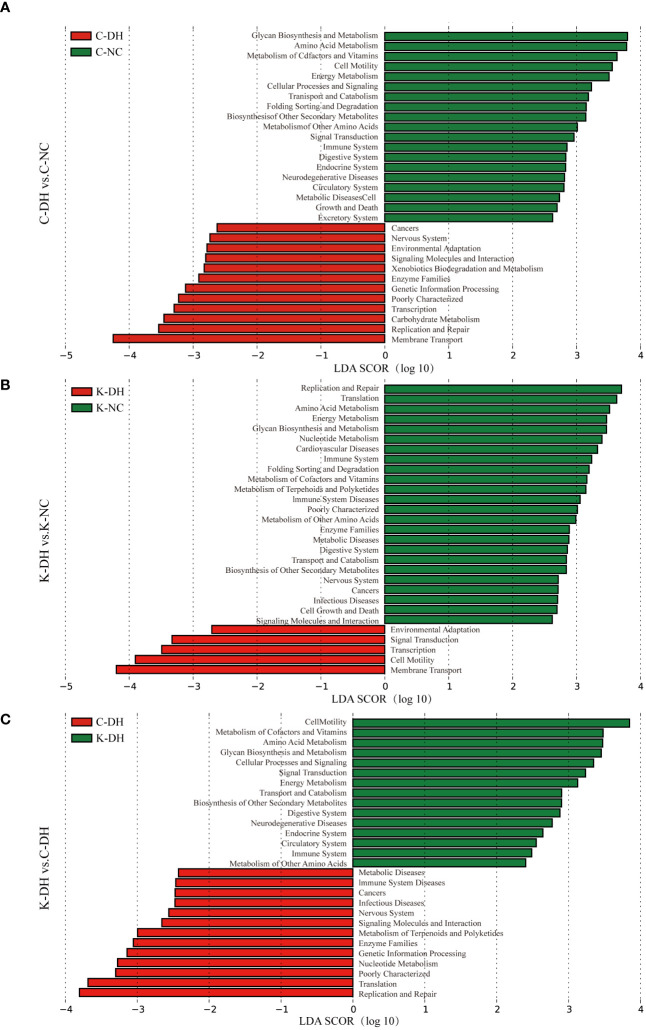
Functional analysis of differential intestinal bacteria. **(A-C)** Enrichment of Kyoto Encyclopedia of Genes and Genomes (KEGG) pathways in mice intestinal microbiota. C57 mice model of humid and hot environment (C-DH). C57 mice in the normal control environment (C-NC). *Nlrp3*-/- mice model of humid and hot environment (K-DH). *Nlrp3*-/- mice in the normal control environment (K-NC).

The Kyoto Encyclopedia of Genes and Genomes (KEGG) of gut microbiota (**Figure 3**) are mainly enriched in eight primary pathways: cellular processes, environmental information processing, genetic information processing, human diseases, metabolism, organismal systems, and two unidentified pathways. Within these eight pathways, 41 secondary pathways were identified. The gut microbiota in the K-DH group was closely associated with pathways related to the digestive system and immune system. The gut microbiota in the C-DH group showed enrichment in pathways related to infectious diseases, tumors, immune system diseases, and metabolic diseases (**Figure 3C**).

### 3.3 The *Nlrp3* promotes humoral immunity and Th2 immune-inflammation in DH modeling

Evaluating the correlation of gene expression level from each group samples to assess the reliability of the experiment and the rationality of sample selection. The correlation coefficient (*R*-value) approaching 1 indicates a higher similarity in gene expression patterns between samples, and all *R* values of the samples in our research are greater than 0.8 (**Supplementary Figure S3**).

**Figure 4:**
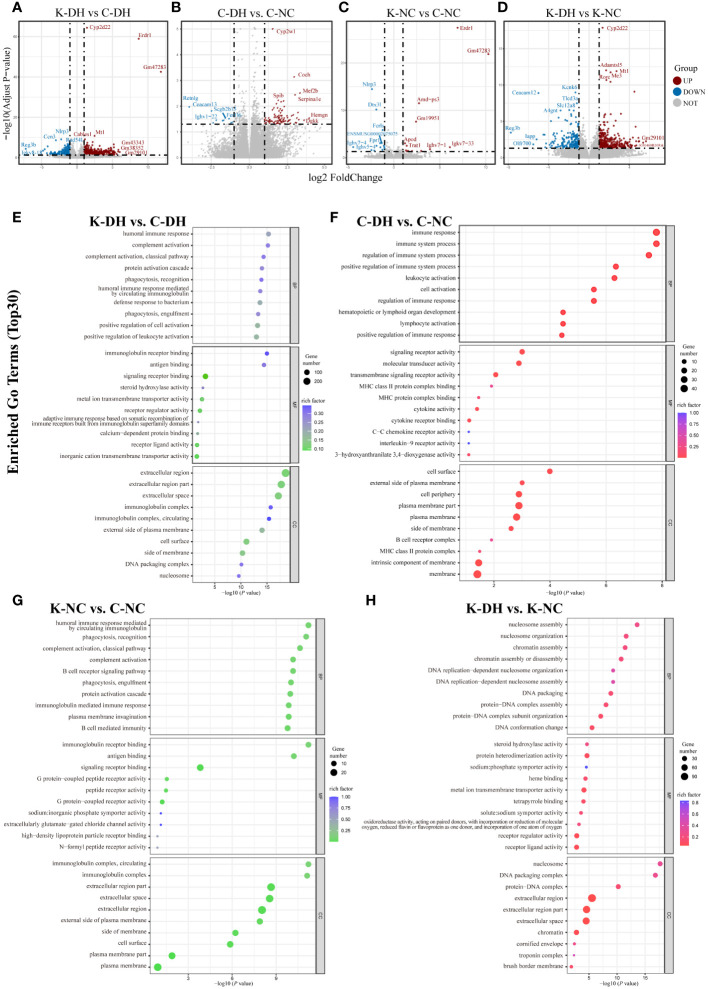
Differential genes and functional analysis in mice transcriptome. **(A-D)** Volcano plot representing differentially expressed genes in mice gut. **(E-H)** Enrichment of GO functions in differentially expressed genes in mice gut. C57 mice model of humid and hot environment (C-DH). C57 mice in the normal control environment (C-NC). *Nlrp3*-/- mice model of humid and hot environment (K-DH). *Nlrp3*-/- mice in the normal control environment (K-NC).

The results of mice intestinal transcriptomics showed that there were 915 genes with significantly altered expression levels in the K-DH group compared to the C-DH group, including 608 upregulated genes and 307 downregulated genes. The expression of genes such as *Ccn3*, *Rad54*, *F2rl3*, and *Kif4* was lower in the K-DH group (**Figures 4A–D**).

The Gene Ontology (GO) enrichment analysis results of differentially expressed genes showed that *Nlrp3* is involved in biological processes such as humoral immune response, complement activation, phagocytosis, recognition, as well as molecular functions such as immunoglobulin receptor binding, antigen binding, and transmembrane transport protein activity (**Figures 4E–H**). The analysis of KEGG enrichment pathways suggest that *Nlrp3* is closely related to malaria, inflammatory bowel disease, type 1 diabetes, JAK-STAT signaling pathway, etc. in DH (**Figure S4A**).

### 3.4 *Nlrp3* deficiency leads to weakened immune function and relatively increased Th1-mediated immunity inflammation

**Figure 5:**
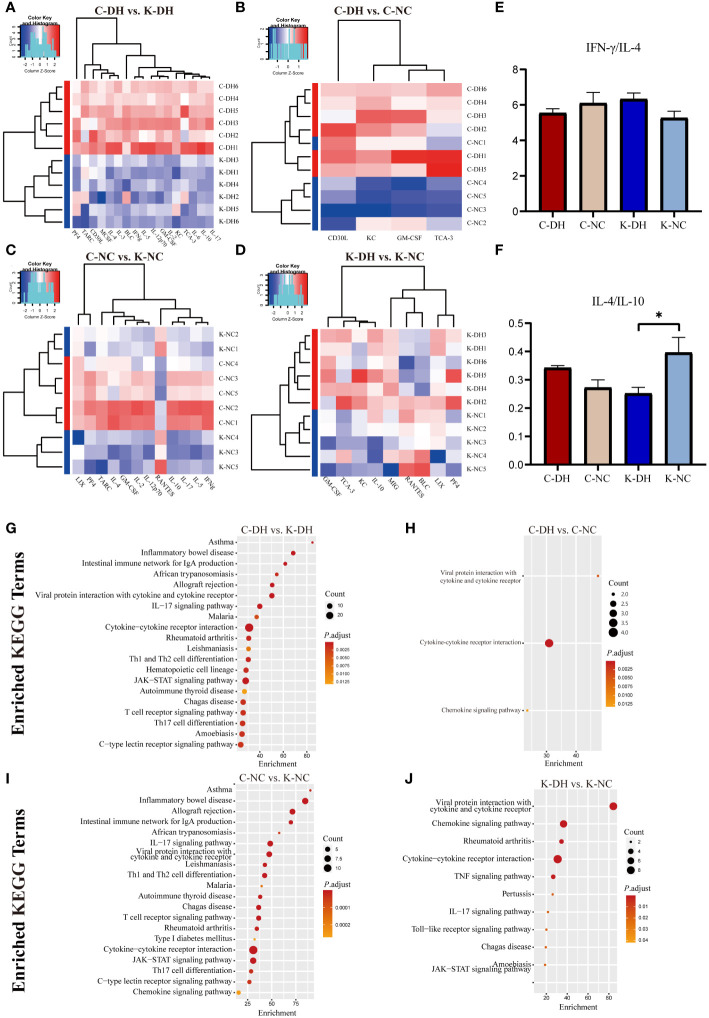
Differential cytokines and analysis function in mice serum. **(A-D)** Principal Component Analysis of serum cytokines in mice. Heatmap clustering of significantly different cytokines in mice serum. **(E, F)** Comparison of IFN-/IL-4 and IL-4/IL-10 ratios in mice serum. **(G-J)** Enrichment of KEGG pathways in differentially expressed cytokines in mouse serum. C57 mice model of humid and hot environment (C-DH). C57 mice in the normal control environment (C-NC). *Nlrp3*-/- mice model of humid and hot environment (K-DH). *Nlrp3*-/- mice in the normal control environment (K-NC). *P < 0.05.

Results as shown by the legend of PCA (**Supplementary Figure S5**), all the clusters marked definitely between groups (**Figures 5A-D**). Compared with the C-NC group, the levels of KC、CD30L、TCA-3 and GM-CSF in the C-DH group were significantly increased (*P*<0.05) (**Figure 5B**). Compared with the C-DH group, the levels of TARC、IL-2、BLC、MCSF、IFN-γ、IL-4、GM-CSF、KC、IL-5、IL-10、IL-6、IL-12 p70、IL-3、CD30L、IL-17、PF4、TCA-3 in the K-DH group were significantly decreased (*P*<0.05) (**Figure 5A**). The comparison of the IFN-γ/IL-4 ratio among the groups only showed a tendency to have more TH1 cytokines of the K-DH (**Figure 5E**), while the IL-4/IL-10 value of the K-DH group significantly decreased compared to the K-NC group (*P*<0.05) (**Figure 5F**).

The KEGG enrichment results showed the association of *Nlrp3* with asthma, inflammatory bowel disease, the intestinal immune network for IgA production, Th1 and Th2 cell differentiation, T cell receptor signaling pathway, IL-17 signaling pathway, JAK-STAT signaling pathway and autoimmune thyroid disease (**Figures 5G–J**).

## 4 Discussion

Gut microbiota is not only a sensor for environmental changes (22) but also an effector that helps the body adapt to environmental changes. The gut microbiota of immigrants will show similar compositional characteristics to local residents (28), and the gut microbiota of mice will show structural differences in different seasons (29). The disruption of the organism’s native gut microbiota structure implies that the colonization resistance of the gut microbiota barrier is compromised, and the susceptibility risk of the organism increases (30).

Previous research results from our research group on the gut microbiota of animal models in humid environments have shown that high humidity leads to a decrease in gut microbiota diversity in mice (31), and DH can also lead to similar decreasing trends (22). In addition, the gut microbiota diversity and abundance of *Nlrp3* ^-/-^ mice are higher (32). In this experiment, the ratio of *Firmicutes/Bacteroidetes* in the C-DH group was increased on the 14th day of the experiment, indicating that the gut microbiota structure of mice was significantly disrupted under the stimulus of a DH (33). The *F/B* ratio of *Nlrp3* ^-/-^ mice was generally lower than that of control mice. In addition, *Proteobacteria* in *Nlrp3* ^-/-^ mice increased slightly as a whole, which may be related to the decrease in *Firmicutes* (34). The C-DH mice overall had fewer *Bacteroidales* (35), which can maintain the gut barrier. The genus *Lactobacillus* in the K-DH group was significantly reduced, indicating that the *Nlrp3* gene may be closely related to the immunosuppressive regulation of Treg cells mediated by gut microbiota (36, 37). It has been found that gut microbiota in *Nlrp3* ^-/-^ and *Asc* ^-/-^ mice were different from that in *IL-18* ^-/-^ and wild-type mice, which suggests that the *Nlrp3* cascade reaction also has different regulatory effects on gut microbiota (38). The abundance results of gut microbiota suggest that DH may promote the immune inflammatory response of the body by affecting the mutual regulation between gut microbiota and *Nlrp3*.

The interaction between the body’s immune response and gut microbiota is an important pathway for maintaining physiological activities. Gut microbiota can regulate the transmission between microbes and hosts through microbe-associated molecular patterns (MAMPs), such as activating TLRs/NLRs pathway-mediated immune inflammatory responses (39). Excessive activation of inflammasomes can cause pathological inflammation, such as NLRP3, NLRC4, NLRP12 and IL-10 (40). Case reports have shown significantly decreased the expression levels of NLRP1, NLRP3, NLRC4, AIM2, and other inflammasomes in patients with colorectal cancer compared to healthy controls (41). These contradictory results suggest that NLRP3 inflammasome plays a complex and multifaceted role in intestinal immune function.

Intestinal tissue macrophages are important gatekeepers for maintaining normal physiological functions. Mucosal macrophages tolerate food antigens and combat bacterial invasion in the digestive tract, while muscle macrophages located in neurons protect gastrointestinal motility (42), all of which are essential for maintaining normal digestive tract function. IL-4 can be produced by activated CD4^+^ T cells, CD4^+^ NK1.1^+^ natural killer T (NKT) cells, group 2 innate lymphoid cells (ILC2s), macrophages, eosinophils, basophils, and mast cells. IL-4 plays a significant role in regulating immune activity and is crucial for the development of Th2-mediated responses (43). Previous studies have found that NLRP3 protein can activate Th2 cells (44) through IL-4 and promote M2 macrophage polarization (45). NLRP3 in dendritic cells is involved in mediating Th2 and Treg cell responses (46) to resist parasitic infections (37). DH modeling may induce dysbiosis in intestinal bacteria, regulate NLRP3 protein to promote TH2-type immunity, and thus polarize local macrophages in the mouse intestine towards M2-type. NLRP3 can also interact with the intestinal microbiota. Through positive regulation of antimicrobial peptides, it can directly induce Treg cells to regulate intestinal tissue immune function.

Th cell-mediated immune response-related cytokines can induce macrophage polarization towards M1 or M2 type, and polarized macrophages also can influence Th cell differentiation (47, 48). Currently, many studies have confirmed the close relationship between NLRP3 inflammasome activation and M1 macrophage polarization (49, 50). Some researchers proposed that NLRP3 can avoid degradation of NLRP3 protein that has not formed inflammasome by binding to interferon regulatory factor 4 (IRF4) (51), and then promote macrophage polarization towards M2 type through IL-4 (52). This provides a pathway and method for NLRP3 to participate in Th2 immune responses. This section’s experimental results suggest that in the context of DH modeling stimulation, mice ileal tissue promotes M2 macrophage polarization through the NLRP3 pathway, and may be involved in promoting Th2 immune differentiation.

Transcriptomic analysis of mouse intestines confirmed the anti-inflammatory effect of *Nlrp3* in DH modeling. For example, there is cross-regulation between *Erdr1* and *IL-18*. Meanwhile, *Nlrp3* inhibiting *Erdr1* may be involved in intestinal inflammation. *Reg3b* can counteract *Salmonella enteritis*, and the decreased level of *Reg3b* in the K-DH group indicates that upregulation of *Nlrp3*-*Reg3b* may be an adaptive protective mechanism against the pathogenic risk of *Salmonella* contamination in DH (53).

Cytokine reflected the close association of *Nlrp3* with inflammation induced by DH, and its important role in Th immune cell differentiation. Compared with C-DH, all significantly changed cytokines in K-DH were reduced, indicating that the overall immune response in *Nlrp3* ^-/-^ mice was restricted. Among the 17 significantly decreased cytokines, 6 cytokines including IFN-γ were involved in Th1 immune response, and 9 cytokines including IL-4 were involved in Th2 immune response. The most pronounced differences in fold change were observed in TARC and IL-2, which promoted the differentiation of Treg cell subsets, suggesting that the Th2 immune response function in the K-DH group was greatly reduced. The proportion of IL-4 in the K-DH group was reduced, indicating that under the stimulation of DH modeling conditions, the mice body might induce a Th2 immune response through *Nlrp3*, leading to an imbalance in the Th1/Th2 ratio. The Th2 immune-promoting role of *Nlrp3* in DH-induced disease deserves attention and exploration.

*Nlrp3*-regulated Th2 immune response not only mediates humoral immunity and inflammatory reactions but also correlates with the differentiation of Treg cells. Treg cells are often generated during the late stage of immune response and inhibit immune responses, which may promote immune tolerance. Engulfing specific apoptotic cells can lead to macrophage reprogramming (54) towards M2 polarization and can inhibit the TLR4/NF-κB pathway, and secrete cytokines such as TGF-β to induce an increase in Treg cells (55), mediating immune suppression.

**Figure 6:**
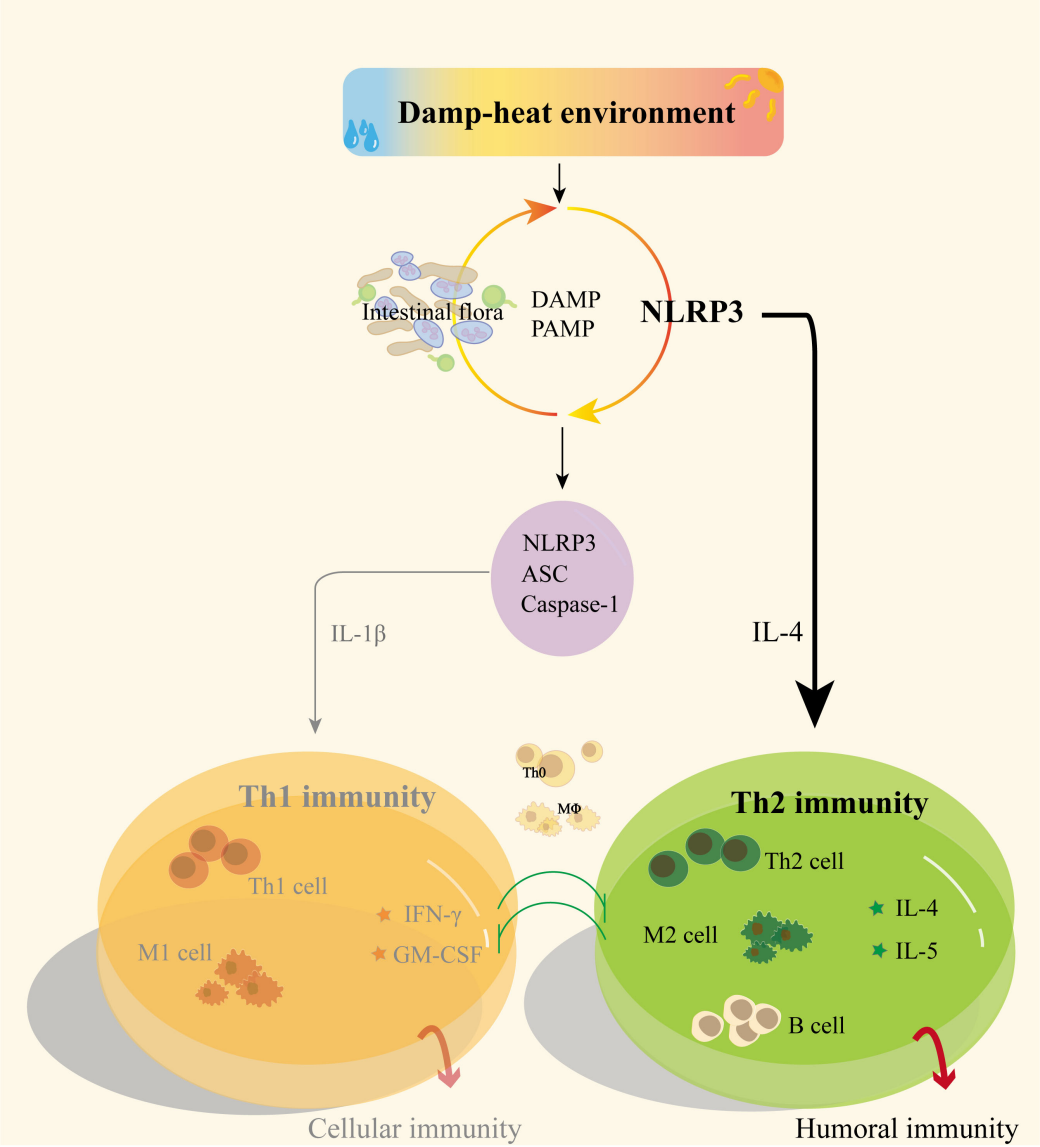
Possible mechanisms of NLRP3 and gut microbiota regulation of Th1/Th2 immunity in DH.

Immune regulatory functions represented by Th1/Th2 balance are of great significance for maintaining the health of the body, and immune balance is the basis for maintaining homeostasis. The Th1 immune inflammatory damage caused by excessive activation of NLRP3 inflammasomes is equally important as the Th2 immune function promoted by *Nlrp3*. Currently, research on NLRP3 mostly focuses on its involvement in Th1 immune response through inflammasome formation, with less attention paid to its Th2 immune role. Taking the Th2 immune regulation function of *Nlrp3* as a starting point may provide new insights into the mechanism of pathogenesis in DH (**Figure 6**).

## Data availability statement

The datasets presented in this study can be found in online repositories. The names of the repository/repositories and accession number(s) can be found below: PRJNA1002631 and PRJNA1001604 (SRA).

## Ethics statement

The animal study was approved by Animal Care & Welfare Committee of Zhongshan Hospital of Chinese Medicine (No. 2022062).

## Author contributions

YL: Data curation, Writing – original draft. XH: Investigation, Writing – review & editing. HH: Writing – review & editing, Software. YW: Methodology, Writing – review & editing. XF: Formal analysis, Writing – review & editing. SC: Writing – review & editing, Formal analysis, Methodology, Project administration. HL: Funding acquisition, Resources, Writing – review & editing, Methodology.
