# Supplementary material for: Intestinal microflora promotes Th2-mediated immunity through NLRP3 in damp and heat environments
Source: Front Immunol. 2024 May 2;15:1367053. doi: 10.3389/fimmu.2024.1367053 (PMC11096527; doi:10.3389/fimmu.2024.1367053)
Supplement: Supplementary file 1 [file DataSheet_1.docx]

Supplementary Materials for

**Intestinal microflora promoting Th2 mediated immunity through NLRP3 in damp and heat environments**

Journal: Frontiers in lmmunology

^*^

*Corresponding author email:

Dr. Huanhuan Luo: avenluo@gzucm.edu.cn

**The PDF file includes:**

Figs. S1 to S5

Tabs. S1 to S2

**SUPPLEMENTARY FIGURES**

**
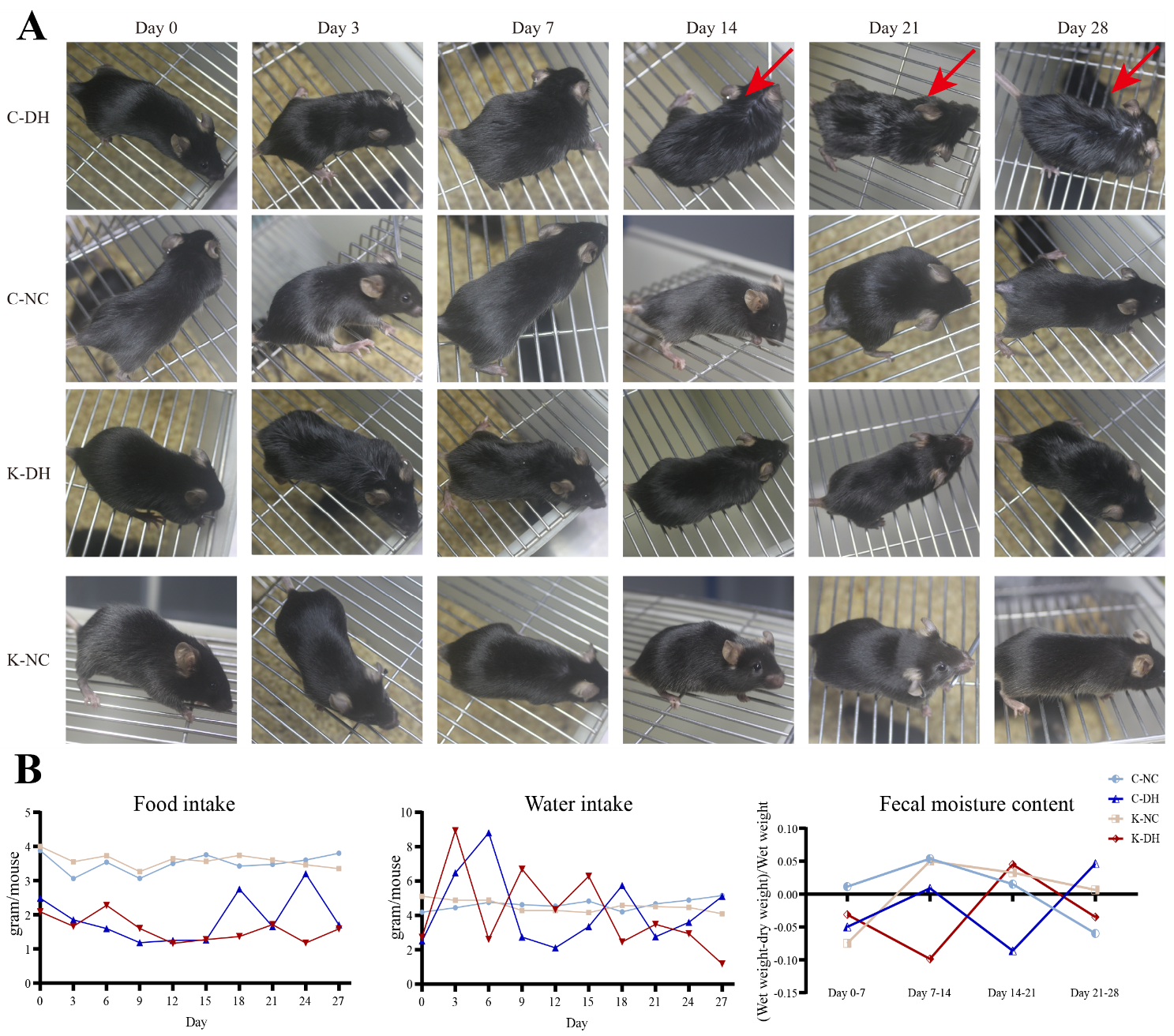
**

**Fig.S1.** **Characterization of changes in mice in a humid and hot environment (DH).**

(A) Degree of mouse fur contamination. (B) Food intake, water consumption, and fecal water content (*mean*). The difference between four groups was not significant.

**
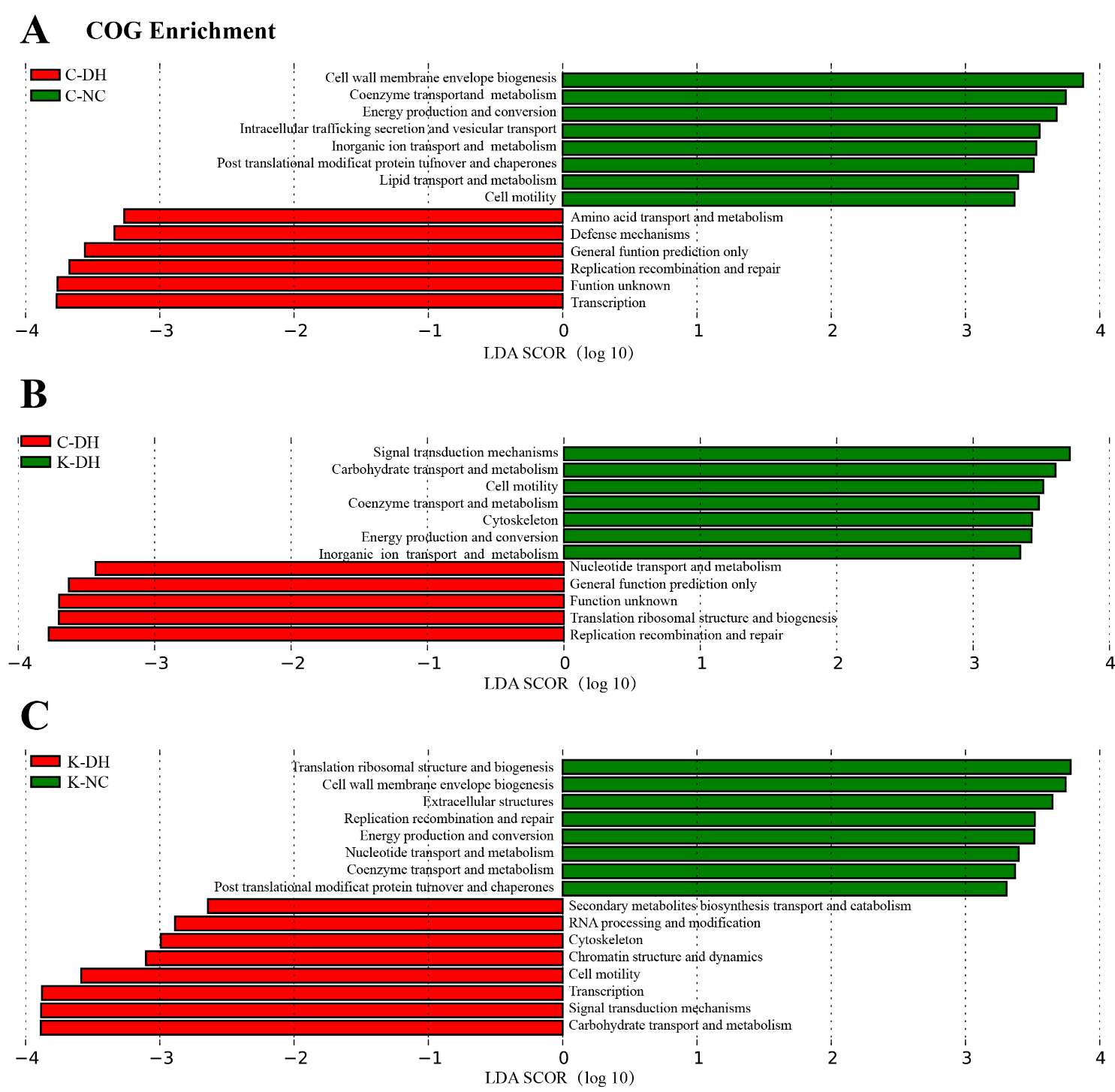
**

**Fig.S2. Cluster of Orthologous Groups (COG) of intestinal bacteria.**

**
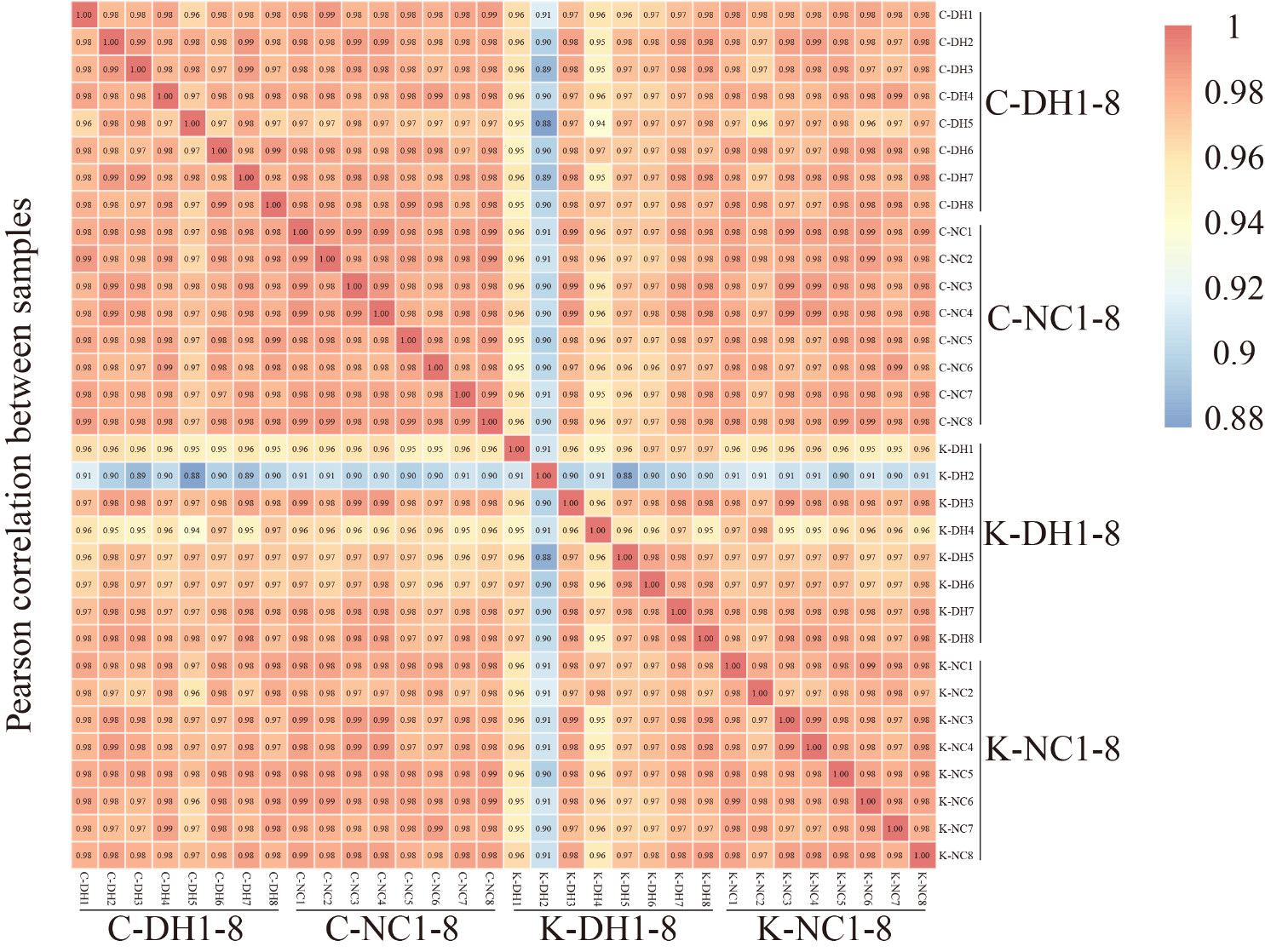
**

**Fig.S3. Effects of modeling a DH on the transcriptome of mice's intestines.**

The correlation coefficient (R value) in gene expression patterns between samples. The horizontal and vertical coordinates in the figure represent the sample numbers, and the numbers represent the R values between two samples. The darker the box color, the higher the correlation. **
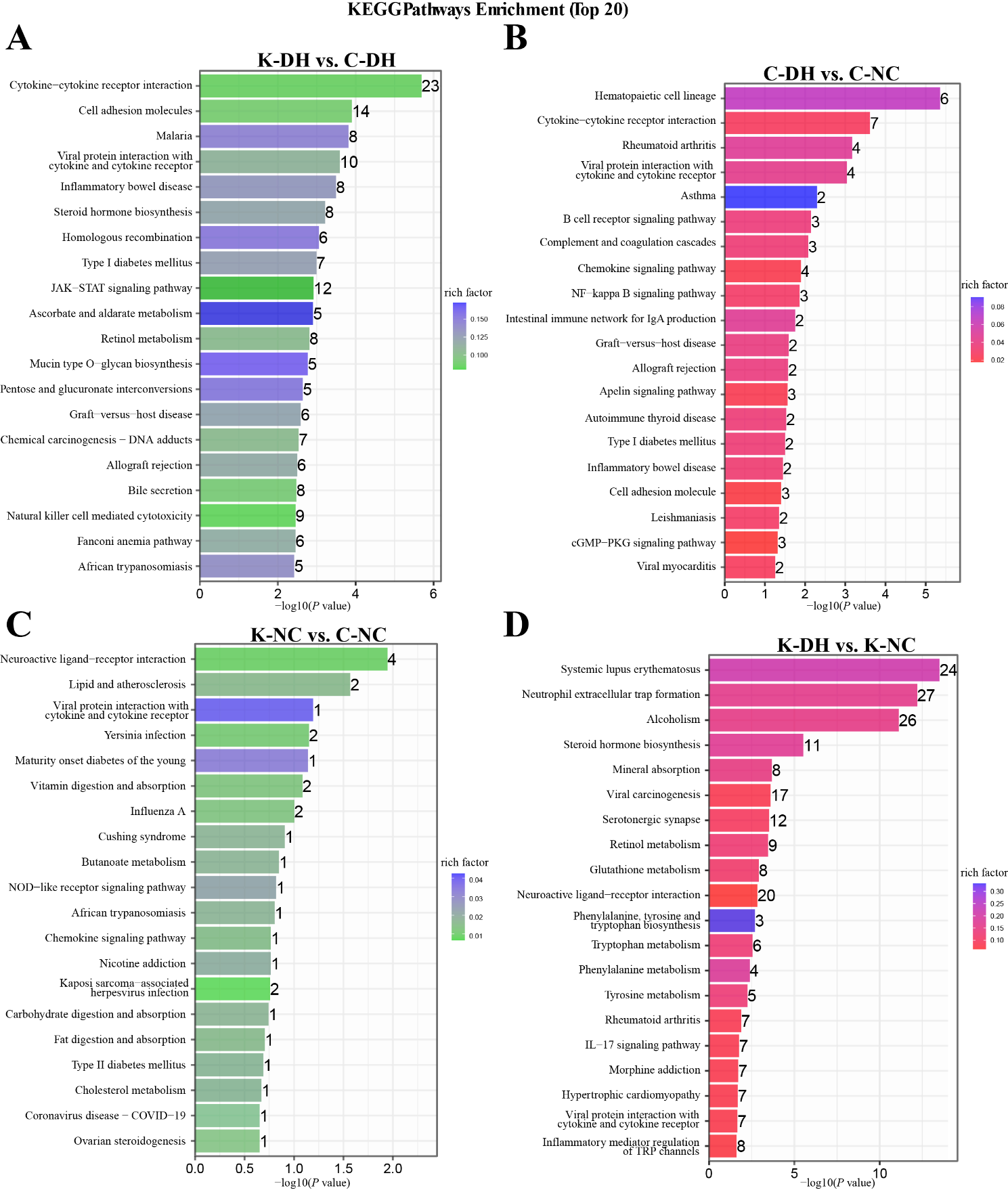
**

**Fig.S4. The KEGG enrichment pathways in differentially expressed genes in mice gut.**

**
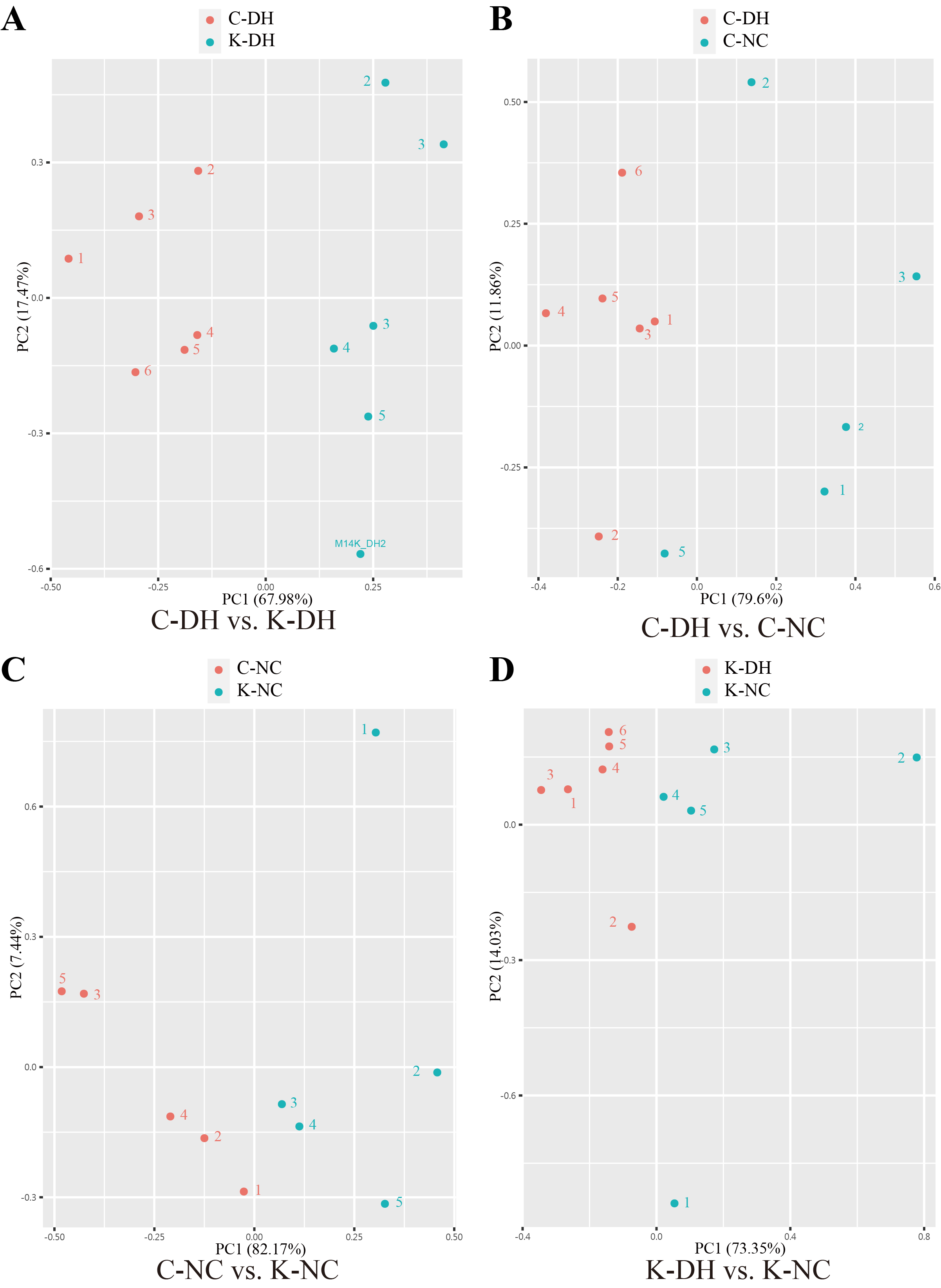
**

**Fig.S5. Distribution of cytokine Principal Component Analysis (PCA).**

**SUPPLEMENTARY TABLE**

**Table 1. Proportions of intestinal bacteria at the Phylum level**

| Phylum | C-DH | C-NC | K-DH | K-NC |
| --- | --- | --- | --- | --- |
| Firmicutes | 83.72% | 28.52% | 47.92% | 30.30% |
| Bacteroidetes | 6.31% | 55.55% | 35.64% | 61.29% |
| Proteobacteria | 1.08% | 3.07% | 3.95% | 4.43% |

Note: C-DH: C57BL/6 mice with damp-heat environment (DH). C-NC: C57BL/6 mice with normal control (NC). K-DH: *Nlrp3*-/- mice with DH. K-NC: *Nlrp3*-/- mice with NC.

**Table 2. Proportions of intestinal bacteria at the Genus level.**

| Genus | C-DH | C-NC | K-DH | K-NC |
| --- | --- | --- | --- | --- |
| Eubacterium  coprostanoligenes group | 0.71% | 0.32% | 0.42% | 0.25% |
| Escherichia-Shigella | 0.01% | 0.01% | 0.04% | 1.06% |
| Proteus | 0.0010% | 0.0000% | 0.0002% | 0.0007% |
| Clostridium | 2.18% | 0.05% | 0.41% | 0.54% |
| Methanobrevibacter | 0.0010% | 0.0017% | 0.0037% | 0.0040% |
| Bacteroides | 0.83% | 0.94% | 4.35% | 2.17% |
